# Characterising co-infections with *Plasmodium spp*., *Mansonella perstans* or *Loa loa* in asymptomatic children, adults and elderly people living on Bioko Island using nucleic acids extracted from malaria rapid diagnostic tests

**DOI:** 10.1371/journal.pntd.0009798

**Published:** 2022-01-31

**Authors:** Charlene Aya Yoboue, Salome Hosch, Olivier Tresor Donfack, Etienne A. Guirou, Bonifacio Manguire Nlavo, Mitoha Ondo’o Ayekaba, Carlos Guerra, Wonder P. Phiri, Guillermo A. Garcia, Tobias Schindler, Claudia A. Daubenberger

**Affiliations:** 1 Department of Medical Parasitology and Infection Biology, Swiss Tropical and Public Health Institute, Basel, Switzerland; 2 University of Basel, Basel, Switzerland; 3 Centre Suisse de Recherches Scientifiques en Côte d’Ivoire, Abidjan, Côte d’Ivoire; 4 Medical Care Development International, Malabo, Equatorial Guinea; 5 Marathon EG Production Ltd, Malabo, Equatorial Guinea; 6 Ministry of Health and Social Welfare, Malabo, Equatorial Guinea; Federation University Australia, AUSTRALIA

## Abstract

**Background:**

Regular and comprehensive epidemiological surveys of the filarial nematodes *Mansonella perstans* and *Loa loa* in children, adolescents and adults living across Bioko Island, Equatorial Guinea are lacking. We aimed to demonstrate that blood retained on malaria rapid diagnostic tests, commonly deployed for malaria surveys, could be used as a source of nucleic acids for molecular based detection of *M*. *perstans* and *L*. *loa*. We wanted to determine the positivity rate and distribution of filarial nematodes across different age groups and geographical areas as well as to understand level of co-infections with malaria in an asymptomatic population.

**Methodology:**

*M*. *perstans*, *L*. *loa* and *Plasmodium* spp. parasites were monitored by qPCR in a cross-sectional study using DNA extracted from a subset malaria rapid diagnostic tests (mRDTs) collected during the annual malaria indicator survey conducted on Bioko Island in 2018.

**Principal findings:**

We identified DNA specific for the two filarial nematodes investigated among 8.2% (263) of the 3214 RDTs screened. Positivity rates of *M*. *perstans* and *L*. *loa* were 6.6% and 1.5%, respectively. *M*. *perstans* infection were more prominent in male (10.5%) compared to female (3.9%) survey participants. *M*. *perstans* parasite density and positivity rate was higher among older people and the population living in rural areas. The socio-economic status of participants strongly influenced the infection rate with people belonging to the lowest socio-economic quintile more than 3 and 5 times more likely to be *L*. *loa* and *M*. *perstans* infected, respectively. No increased risk of being co-infected with *Plasmodium* spp. parasites was observed among the different age groups.

**Conclusions/Significance:**

We found otherwise asymptomatic individuals were infected with *M*. *perstans* and *L*. *loa*. Our study demonstrates that employing mRDTs probed with blood for malaria testing represents a promising, future tool to preserve and ship NAs at room temperature to laboratories for molecular, high-throughput diagnosis and genotyping of blood-dwelling nematode filarial infections. Using this approach, asymptomatic populations can be reached and surveyed for infectious diseases beyond malaria.

## Author summary

*Mansonella perstans* and *Loa loa* are filarial nematodes that infect millions of people living in less developed areas, predominantly in sub-Saharan Africa. Both parasites are neglected among other filarial nematodes because both are regarded as causing mainly asymptomatic infections. The aim of this study was to explore the feasibility of using malaria rapid diagnostic tests (mRDTs) deployed during malaria surveys as a convenient sampling strategy for molecular surveillance of blood-dwelling filarial nematode infections. Our findings demonstrate the potential of mRDTs as a source of parasite DNA beyond malaria, providing an opportunity to expand current knowledge on the distribution and populations mostly affected by *M*. *perstans* and *L*. *loa* infections to Equatorial Guinea, located in Central-West Africa.

## Introduction

Human filariases are vector-borne infectious diseases that encompass Mansonellosis and loiasis [1]. Mansonellosis is caused by three main nematode species, *M*. *perstans*, *M*. *streptocerca* and *M*. *ozzardi* [2]. Recently, an additional species has been described in Gabon named *Mansonella sp* "DEUX" [3]. Mansonellosis is one of the most neglected tropical diseases despite the fact that in large parts of sub-Saharan Africa, as well as in Latin America an estimated 100 million people are infected [2,4]. The life cycles of *Mansonella* spp. generally alternate between an insect vector and humans who are the final hosts. The insect vectors transmitting *M*. *perstans* belong to the genus *Culicoides* [2]. When feeding on an infected human, female vectors pick-up microfilariae (mf) circulating in the blood. The mf penetrate the insect´s gut and undergo developmental stages in the thoracic flight muscle, migrate to the head and proboscis from where *M*. *perstans* is transferred to humans during the next feeding round [2]. The third-stage infective larvae (L3) actively penetrate the human skin before migrating and maturing into adult worms that can be found in serous body cavities, mainly the peritoneal cavity [2]. Adult male and female worms mate and female worms begin to produce unsheathed mf circulating in peripheral blood. Unsheathed mf of *M*. *perstans* are detected by microscopic examination in thick or thin blood films stained with Giemsa in blood samples taken at any time of the day [5,6]. Little is known about the clinical outcome of chronic *M*. *perstans* infections in endemic populations, and as for other filarial infections most infections seem to be clinically silent [2,5]. Clinical symptoms attributed to *M*. *perstans* infections include eosinophilia, angioedema, arthralgia, fever, headache, pruritus, skin eruption, serositis, neurologic manifestations, ocular or palpebral pruritus, visual impairment and chest pain [1].

At least 10 million people are infected with *Loa loa* in endemic countries of Central, and Western Sub-Saharan Africa [7]. *L*. *loa* larvae are transmitted to humans during blood feeding of an infected vector fly belonging to the genus *Chrysops* spp. [1]. The sheathed adult worms live freely in the subcutaneous tissues where they produce thousands of sheathed mf daily, usually with a peak between 10 AM and 4 PM [8]. Diagnosis of *L*. *loa* can be challenging since adult worms can be present without detectable mf in blood [8].

In areas where different filarial nematodes are co-endemic, misclassification of mf by microscopy can be problematic [8,9]. Therefore, molecular methods have been developed to improve filarial nematode detection [10] and qPCR-based molecular techniques have shown higher sensitivity to detect low parasite density infections, and to discriminate between different filarial nematode species [8]. Large-scale implementation of molecular diagnostic methods for neglected tropical diseases has been regarded as a challenge in the public health systems of low and middle-income countries based on cost, human resource requirement and complexity of supply chain management [11].

The aim of this work was to demonstrate that blood retained on malaria rapid diagnostic tests (mRDTs) is a source of nucleic acids for molecular based detection of *M*. *perstans* and *L*. *loa* in otherwise asymptomatic individuals. In doing so, we described the association of filarial infections with demographic and geographic factors and assessed the level of multi-parasitism of these nematodes with the highly endemic malaria parasites on Bioko Island. We also used Cq values as a proxy of filarial parasite density and compared this measurement against demographic and geographic characteristics of the investigated population as well as the time of the day of RDT sample collection.

## Material and methods

### Ethics statement

The MIS in 2018 was approved by the Ministry of Health and Social Welfare of Equatorial Guinea and the Ethics Committee of the London School of Hygiene & Tropical Medicine (Ref. No. LSHTM: 5556). Consent was sought from eligible respondents before the administration of the questionnaire. A signed authorization was requested from the parents or legal guardians of children, and adult participants to use their samples for further analyses. Laboratory experiments were performed in accordance with relevant guidelines and regulations.

### Study area

Bioko Island is located on the West African continent shelf, precisely in the Gulf of Guinea and separated from Cameroon by no more than 32 kilometres of shallow ocean. With its total land surface of approximately 2000 km^2^, Bioko forms part of the nation of Equatorial Guinea and is administratively divided into four districts: Malabo, the capital of Equatorial Guinea, and Baney both located in the northern part of the Island; and Luba and Riaba both located in the Southern part. The Island has an estimated population size of 270´000 people, with the majority (85%) living in Malabo [12]. Bioko has a typical equatorial climate, with high temperatures, high humidity, and heavy rainfall [13].

Malaria has historically been hyper-endemic in Bioko Island with a prevalence of 45% among 2–14 year old children before the launch of the Malaria control program [14,15]. The Bioko Island Malaria Control Program—implemented since 2003—has successfully reduced malaria prevalence and malaria related morbidity and mortality [14,16]. Malaria indicator surveys (MIS) have been conducted annually since 2004 within sentinel sites across the island to enable monitoring and evaluation of this programme [14,17].

### Study design

The 2018, the MIS on Bioko Island was conducted on a representative sample using the Malaria Pf/PAN (HRP2/pLDH) Ag Combo rapid diagnostic test (ACCESS BIO, NJ, USA). Consent to store used mRDTs for further molecular analyses was obtained from 13,505 survey participants and unique barcode labels were affixed to the used mRDTs that were shipped to the Swiss Tropical and Public Health Institute for further analyses. Each mRDT barcode was linked to a household unique identification code [18]. This allowed a detailed geographic allocation of the mRDT results. The age distribution of MIS 2018 participants differed slightly between the four districts. Malabo district with median age of 17 years, (IQR 7–30) and is characterized by a slightly younger MIS 2018 population than Riaba (17 years, IQR 7–40), Luba (19 years, IQR 8–45) or Baney (16 years, IQR 7–32). To derive parasite positivity rate estimates, all individuals found positive and all tested in the sample were aggregated at a 1x1 km^2^ grid. Coinfections were estimated at the same spatial resolution, for comparison.

A subset of the mRDT were selected for molecular analysis of the blood retained on the mRDT based on the mRDT test outcome for malaria. Out of the 1376 malaria positive mRDTs identified during the 2018 MIS, we analysed the nucleic acids from 1065 mRDTs (77.4%). In addition, other malaria negative mRDT were selected for the molecular analysis.

### Extraction of nucleic acids from used mRDTs

We reused the extracted nucleic acids (NAs) from a study which was published recently by our group [19]. Briefly, the entire and uncut nitrocellulose strip in the used mRDT was carefully removed and incubated in a lysing buffer at 60°C for two hours. After several washing steps, NAs were eluted in a final volume of 75 μL and stored at -20°C. The extracted NAs were amplified and detected by reverse transcription quantitative real time PCR (RT-qPCR) to identify and quantify *Plasmodium* spp. parasites [19]. For the study presented here, we extracted and analysed NAs from additional mRDTs collected during the same MIS and extend the approach by using the extracted NAs to detect the filarial nematodes, *M*. *perstans* and *L*. *loa*, by qPCR using well described marker genes [20,21]. We have calculated the median number of days between blood collection onto the mRDT during the MIS 2018 and extraction of NA in our laboratory in Basel to be 253 days (IQR 138–352 days).

### *Loa loa* and *Mansonella perstans* detection by a multiplex qPCR assay

A multiplex qPCR assay, herein referred to as llmp-qPCR, was developed and performed to detect *L*. *loa* and *M*. *perstans* DNA. In a multiplex qPCR reaction, the *L*. *loa* specific qPCR assay amplifies a 62 base pair (bp) fragment from the hypothetical protein LLMF72 [20] and the *M*. *perstans* specific qPCR assay is based on amplification of a 187 bp fragment of the 18S ribosomal RNA gene [21]. The human rnasep gene (RNaseP) served as an amplification internal control to monitor the successful extraction and amplification of DNA [22]. All reactions were run in duplicates on 96-well plates. Molecular biology grade H_2_O was run as a non-template control, and a mix of *M*. *perstans* and *L*. *loa* DNA served as a positive control for each run. Each reaction contained 2 μL of template DNA and 8 μL master mix consisting of 1 x Luna Universal Probe qPCR Master Mix (New England Biolabs, Ipswich), 0.4 μM of *M*. *perstans* forward primer, 0.4 μM of *M*. *perstans* reverse primer, 0.25 μM of the Yakima Yellow-labelled *M*. *perstans* probe, 0.8 μM of *L*. *loa* forward primer, 0.8 μM of *L*. *loa* reverse primer, 0.4 μM of FAM-labelled *L*. *loa-*specific minor groove binder (MGB) probe, 0.4 μM HsRNaseP forward primer, 0.4 μM of HsRNaseP-reverse primer and 0.4 μM of Cy5-labelled HsRNaseP probe. Using the Bio-Rad CFX96 Real-Time PCR System (Bio-Rad Laboratories, California, USA), amplification program was 1min at 95°C, followed by 50 cycles of 15s at 95°C and 45s at 55°C. Samples were considered positive if the quantification cycle (Cq) value was <50.

### Co-infection of *Mansonella perstans* and *Loa loa* with *Plasmodium* spp.

The PlasQ is a multiplex RT-qPCR assay for *Plasmodium* spp. and *P*. *falciparum* detection and quantification that has been developed by our group and described previously [19,22]. This qPCR assay consisted of amplification of two targets combined in a multiplex assay, namely the Pan-*Plasmodium* 18S rDNA sequence (Pspp18S) and the *P*. *falciparum*-specific acidic terminal sequence of the var genes (PfvarATS). The human RNaseP (HsRNaseP) gene served as an internal control to assess the quality of DNA extraction and qPCR amplification. The PlasQ was performed on NA extracted from mRDT and samples with Cq value <45 of either of the two targets, PfvarATS or Pspp18S, were considered positive for *Plasmodium* spp. Then, results obtained were linked to llmp-qPCR results obtained from the same aliquot of NA extracted from identical mRDT to assess co-infection status between Plasmodium spp., *L*. *loa* and *M*. *perstans*. Coinfections were estimated at the same 1x1 km^2^ grid, for comparison.

### Sanger sequencing analysis of *Mansonella perstans* and *Loa loa*

The ribosomal internal transcribed spacer 1 region was amplified with a set of primers that bind universally to all filarial species and are designed to highlight interspecific differences [23]. The PCR products were 484 base pairs (bp) for *M*. *perstans* and 457 bp for *L*. *loa*. PCR products of 10 and 23 samples tested positive by llmp-qPCR for *L*. *loa* and *M*. *perstans*, respectively, were sequenced from both ends by Sanger Sequencing (Microsynth AG, Balgach, Switzerland) to confirm specificity of the qPCR assays. Samples covering a large and representative range of different Cq values were selected for sanger sequencing of the ribosomal internal transcribed spacer 1 region (S4 Fig). Sequence analysis was realized using Geneious Prime 2019.1.1 (https://www.geneious.com). A consensus sequence of all 23 *M*. *perstans* and 10 *L*. *loa* sequences of 417 bp and 390 bp length, respectively, served as a query sequence to identify all GenBank entries with >90% identity and >95% coverage using BLAST. Additionally, representative sequences for *M*. *streptocerca* (KR868771) and *M*. *ozzardi* (EU272180) deposited to GenBank were included. Geneious Prime software (version 2021.0.3) was used for the multiple sequence alignment and to generate the phylogenetic analysis using its in-build neighbour-joining (NJ) clustering method [24]. Branch lengths were estimated with the Tamura-Nei model [25] with *Onchocerca volvulus* (EU272179) as an outgroup. The resulting newick file was imported into R for final phylogeny and visualization using the ggplot2, ggtree, and treeio packages.

### Data analysis

Households were assigned scores based on the type of assets and amenities they own (radio, television, sofa, fan, air-conditioner, car, etc) to derive a surrogate of Socio-Economic Status (SES), using Principal Component Analysis (PCA). Households were ranked based on their score and the distribution was further divided into five equal categories (quintiles), each with approximately 20% of the households. The first quintile corresponded to the lowest wealth index (WI) and the fifth to the highest WI. The household WI categories were also assigned to permanent (de jure) household members. Predicted co-infection rates for *Plasmodium* spp. and *M*. *perstans* or *L*. *loa* were expressed as the product and 95% confidence interval (95% CI) of *Plasmodium* spp., *M*. *perstans* and *L*. *loa* the prevalence stratified by age group. The ELIMU-MDx platform was used for quality control, management and analysis of qPCR data [26]. Statistical analysis and visualization of data were conducted using R version 3.5.1. Univariate analysis (Fisher’s exact test and Wilcoxon-Mann-Whitney-Test, as appropriate) was used for comparison between groups. P***-***value < 0.05 was considered statistically significant.

## Results

### Sample selection and study population characteristics

A total of 4774 households, including 20’012 individuals, from across Bioko Island participated in the MIS 2018. 13’505 participants provided an additional consent for molecular analysis of the mRDT collected. To increase the probability to identify individuals with filarial nematodes and *Plasmodium* spp. co-infections, we over-sampled mRDTs from two specific sub-populations. Firstly, malaria positive mRDTs were preferentially selected and processed and secondly, for filarial nematodes infections, mRDTs from adults living in rural districts were enriched for selection and analysis. A graphical representation of the over-sampling is shown in S1 Fig. Among the mRDTs selected for NAs extraction, 1065 mRDTs were malaria positive, accounting for 75.8% of all positive mRDTs identified during the 2018 MIS. Significantly higher proportions of adults and people living outside of urban Malabo were included. The subset of mRDTs which were selected to investigate the positivity rates of *M*. *perstans* and *L*. *loa* infections stratified by geographical location, age and socio-economic status is shown in Table 1. Noteworthy, from each district or age group at least 10% of the collected mRDTs were included into the analysis. In summary, the majority of the samples included were collected in Malabo (64%). The proportion of mRDTs collected from women was higher compared to men. The mean age was 22 years (SD = 19.7) and participants aged <20 belonged to the most common age group (45.4%). Socio-economic status was higher in participants living in Malabo and Baney compared to the two southern districts (Luba and Riaba). The mean haemoglobin value of all participants was 12.02 g/dl (SD = 1.9) and 99.4% of people did not have fever at time of the sample collection.

**Table 1:** Distribution of population included by age, gender, sociodemographic status and district of residence.

Characteristics	*Malabo (n =* 2064)	Baney (n = 690)	Luba (n = 257)	Riaba (n = 203)	Total (n = 3214)
**Gender**					
Women (%)	1261 (61.1)	384 (55.7)	139 (54.1)	100 (49.3)	2086 (55.0)
Men (%)	803 (38.9)	306 (44.3)	118 (45.9)	103 (50.7)	1704 (45.0)
**Age (years)**					
0–19 (%)	1261 (61.1)	110 (15.9)	42 (16.4)	47 (23.2)	1460 (45.4)
20–39 (%)	576 (27.9)	379 (54.9)	58 (22.7)	46 (22.7)	1059 (33)
40–59 (%)	111 (5.4)	141 (20.4)	81 (31.6)	58 (28.6)	391 (12.2)
≥ 60 (%)	116 (5.6)	60 (8.7)	75 (29.3)	52 (25.6)	303 (9.4)
**Socio-economic status (quintile)**					
1 lowest	185 (9.0)	153 (22.5)	93 (36.3)	94 (46.3)	525 (16.4)
2 second lowest	348 (16.8)	132 (19.4)	66 (25.8)	53 (26.1)	599 (18.7)
3 middle	473 (22.9)	130 (19.1)	29 (11.3)	32 (15.8)	664 (20.7)
4 second highest	487 (23.6)	119 (17.5)	49 (19.1)	22 (10.8)	677 (21.1)
5 highest	571 (27.7)	145 (21.4)	19 (7.4)	2 (1.0)	737 (23.0)

### Positivity rates of *L*. *loa* and *M*. *perstans* among participants of the annual malaria indicator survey

**Fig 1:**
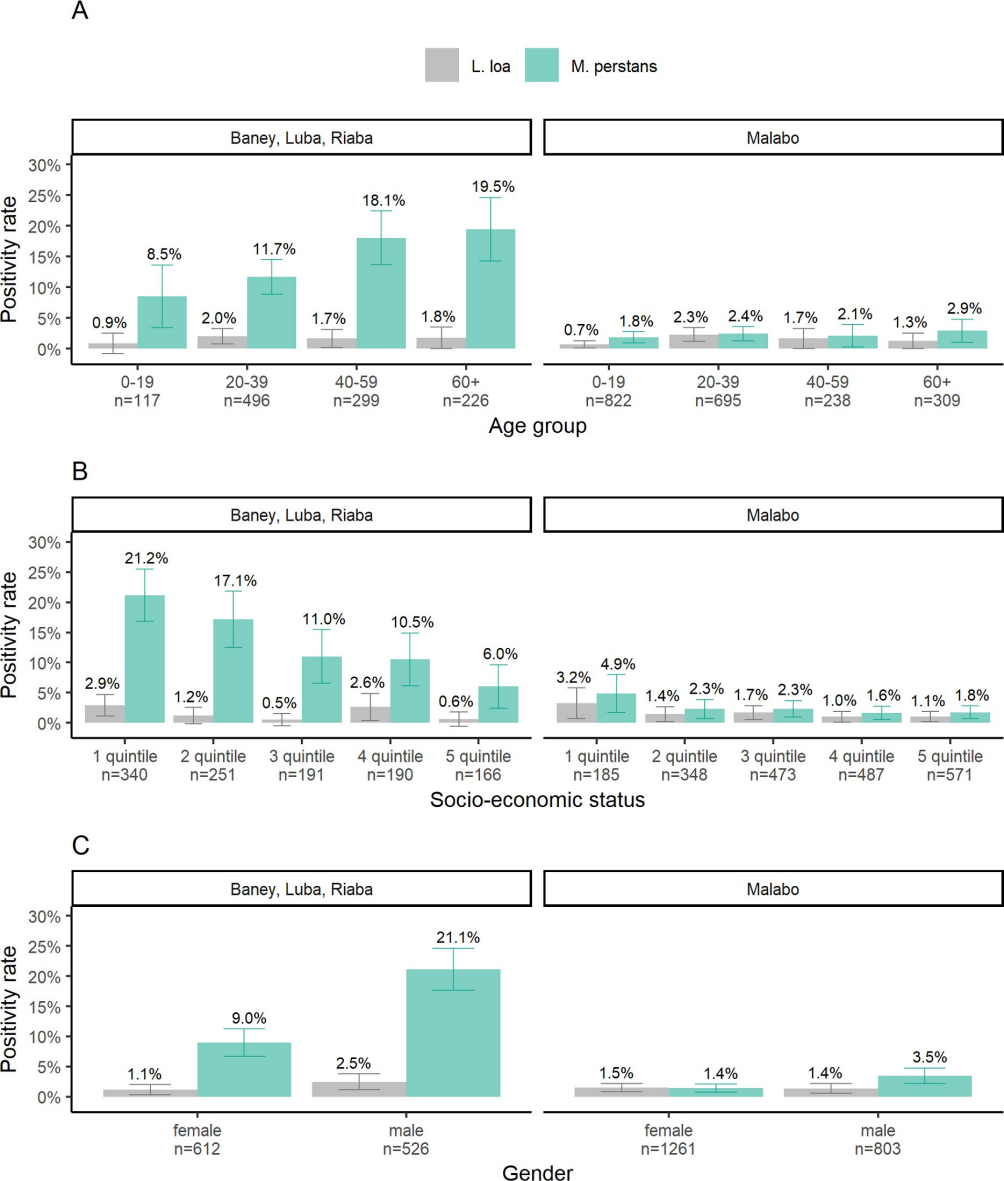
Positivity rates of *M*. *perstans* and *L*. *loa* by rural and urban districts. (A) across all age groups investigated, (B) grouped according to socio-economic status and (C) stratified by gender. Positivity rates and 95% confidence intervals were calculated as the proportion of llmp-qPCR positive mRDTs in all tests carried out in each group and are given on top of each bar. Data supporting the Fig 1 are detailed in S2 Table.

Using the llmp-qPCR assay, detecting simultaneously *M*. *perstans* and *L*. *loa* in a single qPCR reaction (S2 Fig), of the 3214 mRDTs that were tested, 8.2% (263) were positive for *M*. *perstans* and/or *L*. *loa*. The proportion of mRDTs positive for *M*. *perstans* was 6.6% (213) compared to 1.5% (50) for *L*. *loa*. Fig 1 details the positivity rates of *M*. *perstans* and *L*. *loa* stratified by age (A), socio-economic status (B) and gender (C). People living in rural districts have significantly higher positivity rates for *M*. *perstans* than people living in the urban areas. Positivity rates in rural districts ranged from 17.1% (Luba) to 13.2% (Baney) compared to 2.1% in the urban district of Malabo. On the contrary, no significant differences in *L*. *loa* positivity rates were observed between rural and urban districts. *L*. *loa* was more prevalent in the two Southern districts, Riaba (3.9%) and Luba (2.7%), compared to the Northern districts of Malabo (1.5%) and Baney (0.7%). *M*. *perstans* infection rates in high-endemic rural districts increased significantly with age and the highest positivity rate was observed in participants older than 60 years (*p*< 0.00001). *L*. *loa* was found at higher rates in participants older than 20 years of age in urban as well as in rural areas (*p* = 0.0001) (Fig 1A). Among children below the age of five, a positivity rate of 1.4% (4/296) for *M*. *perstans* and not a single infection for *L*. *loa* was observed. In older children and adolescents, positivity rates of 2.4% and 0.7% were estimated for *M*. *perstans* and *L*. *loa*, respectively. Infection rates were strongly influenced by the socio-economic status of the individuals (Fig 1B). People from rural district assigned to the lowest SES were three times more likely to harbour an *M*. *perstans* infection than people belonging to the highest SES. The same was observed for *L*. *loa* where the positivity rate was also higher among lowest SES compared to highest SES.

While the proportion of *L*. *loa* infections was comparable between male and female individuals combined for rural and urban districts (*p* = 0.4), *M*. *perstans* was significantly higher among male (21.1%) compared to the female (9.0%) inhabitants of rural districts (*p*< 0.00001) (Fig 1C).

### Filarial nematode species identification by ribosomal internal transcribed spacer 1 region sequence analysis

**Fig 2:**
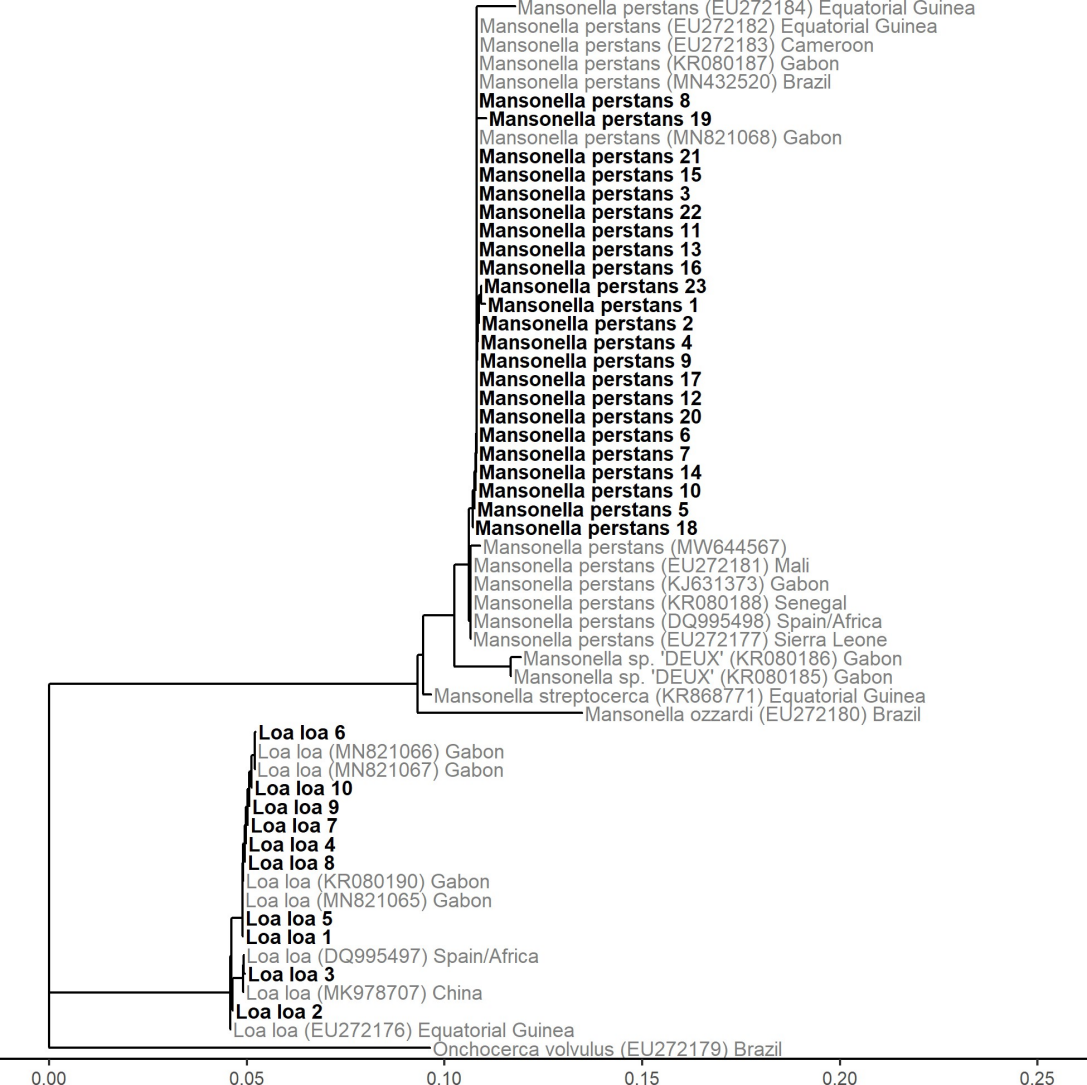
Phylogenetic tree of the ribosomal internal transcribed spacer 1 region of *M*. *perstans* and *L*. *loa*. PCR products were amplified from DNA extracted from mRDTs and sequenced. The scale on the x-axis corresponds to the number of substitutions per site (number of changes/sequence length).

Specificity of the qPCR-based species identification was confirmed by sequence analysis of the conserved ribosomal internal transcribed spacer 1 region that encompassed 390 to 450 bp depending on the filarial species (Fig 2). To our knowledge, this is the first time that a molecular marker, regularly used for filarial nematode species identification, was amplified and sequenced from DNA extracted from blood retained on mRDTs. Twenty-three samples positive for *M*. *perstans* and ten samples positive for *L*. *loa* identified by the llmp-qPCR assay were all confirmed by sequence analysis. The *M*. *perstans* sequences clustered closely with each other and other *M*. *perstans* sequences, but are distinct from *Mansonella* spp. DEUX sequences. The *L*. *loa* sequences generated in this study are closely related to sequences from Central- and West-Africa deposited in GenBank.

### Geographical distribution of *M*. *perstans*, *L*. *loa* and *Plasmodium* spp. show distinct patterns

**Fig 3:**
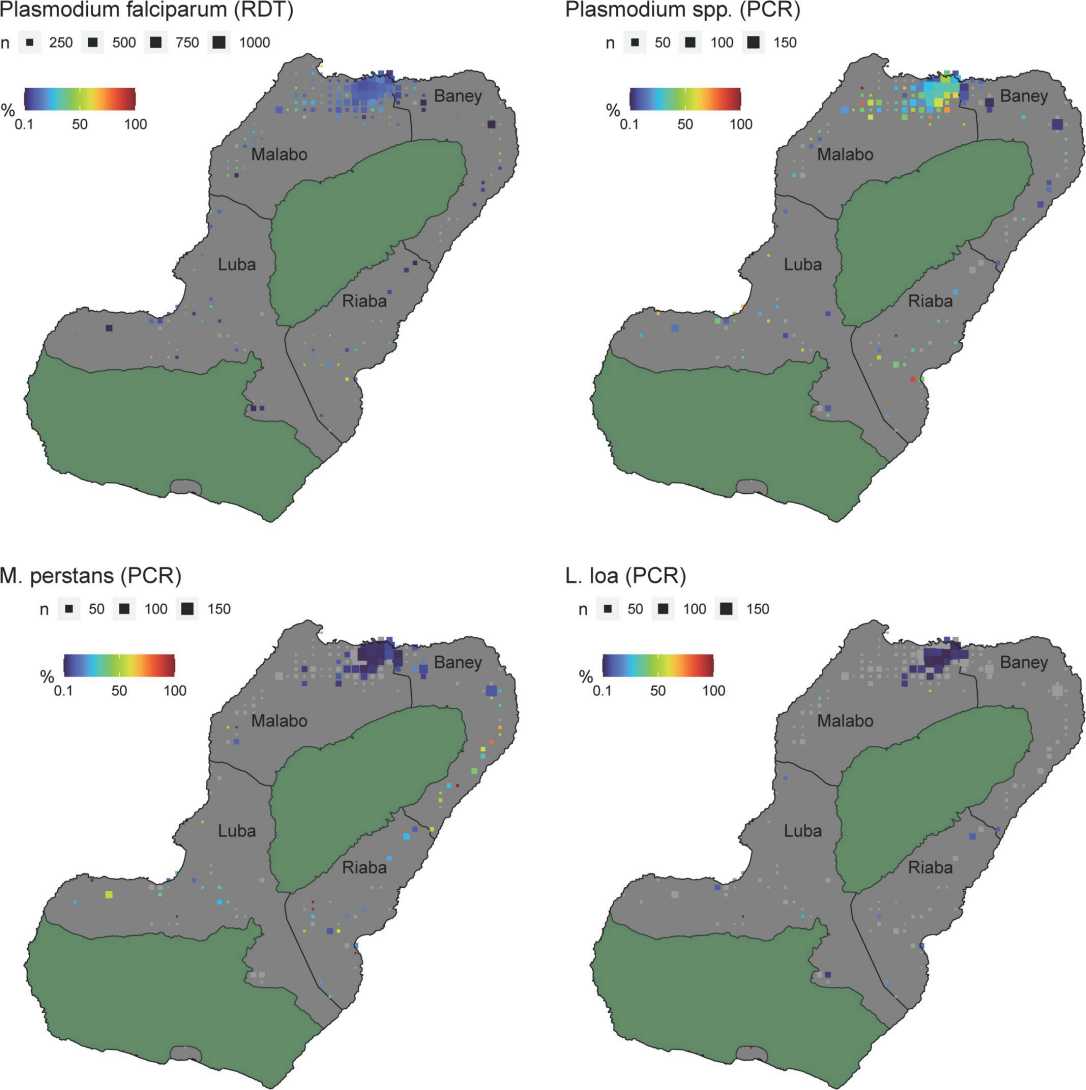
Positivity rate of *Plasmodium* spp, *M*. *perstans* and *L*. *loa* across Bioko Island. (A) Prevalence of PfHRP2-positive mRDTs for the entire MIS 2018 population. (B) *Plasmodium* spp. RT-qPCR positivity rate of mRDTs selected for molecular analysis. (C) *M*. *perstans* qPCR based positivity rate. (C) *L*. *loa* qPCR based positivity rate. The size of the squares represents the number of people analysed living in the corresponding 1x1 km^2^ grid. The areas marked in green are nature reserves. Greyed out spots on the maps represent settlements from which mRDTs were collected with null positivity rate for Plasmodium *spp*, *M*. *perstans* and *L*. *loa*.

On Bioko Island the environmental living conditions for the population differ starkly between the urban centre in Malabo and the rural districts in Baney, Riaba and Luba. Therefore, we mapped the prevalence and geographical distribution of *P*. *falciparum* for all samples collected during the MIS based on mRDT positivity in Fig 3A. To investigate the level of co-infections between malaria and filarial parasites in different populations, malaria positive RDTs were given priority when selected for molecular analysis described here. This intentional enrichment resulted in higher positivity rates of *Plasmodium* spp. when analysed by RT-qPCR (Fig 3B). High positivity rates of *M*. *perstans* were found in areas along the East coast as well as in the Southern districts; while in urban areas around Malabo, *M*. *perstans* was found at low rates or was even absent (Fig 3C). *L*. *loa* positivity rates are generally low and no distinct patterns are seen. Interestingly, no *L*. *loa* were found in the district of Baney, where positivity rates of *M*. *perstans* are the highest (Fig 3D).

### Co-infection of *M*. *perstans* or *L*. *loa* with *Plasmodium* spp. parasites

**Fig 4:**
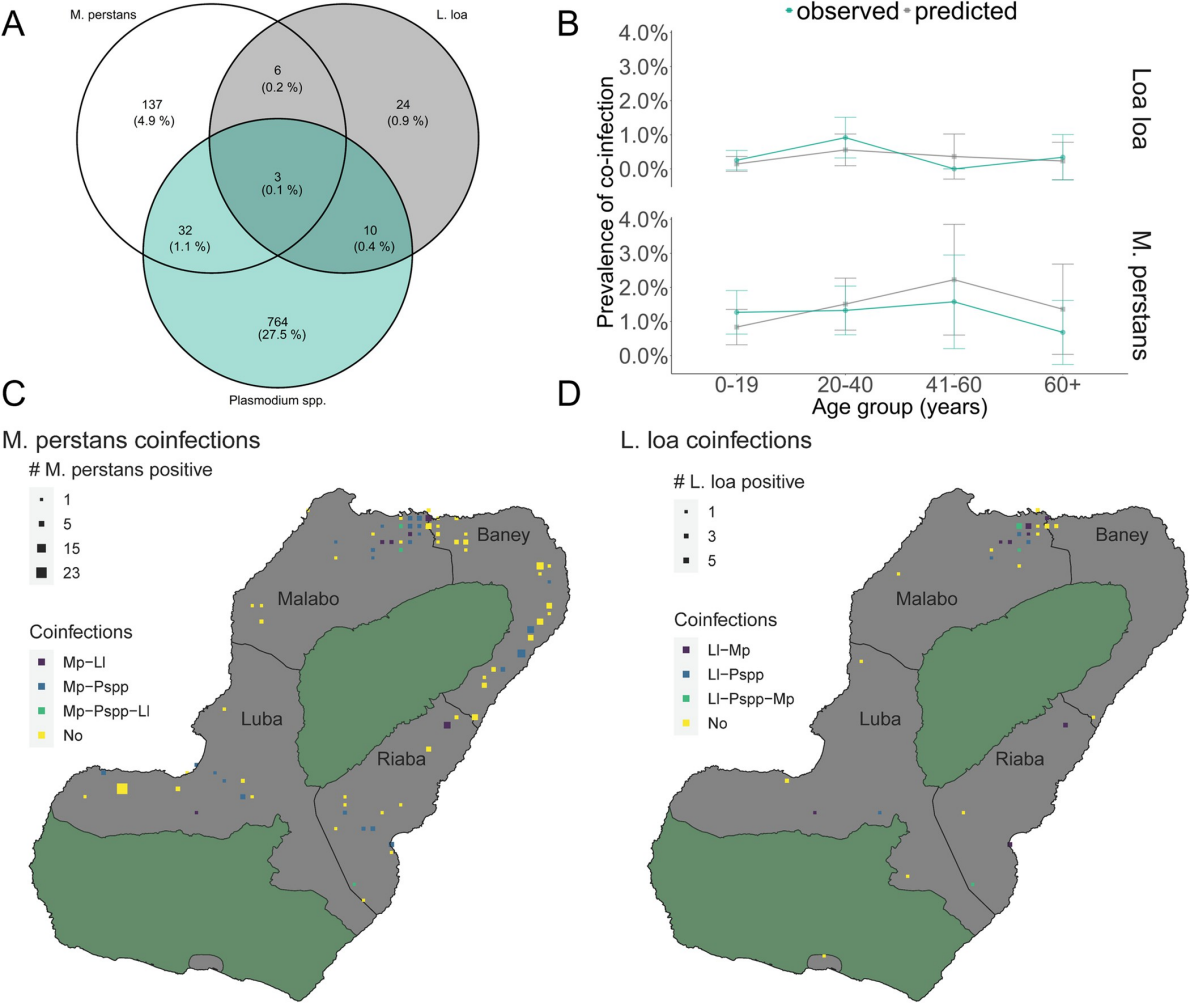
*Plasmodium* spp., *M*. *perstans* and *L*. *loa* multi-parasitism infections on Bioko Island. (A) Number of *M*. *perstans*, *L*. *loa* and *Plasmodium* spp. co-infections amongst 2775 individuals tested. (B) Positivity rates of *Plasmodium* spp., *M*. *perstans* and *L*. *loa* co-infections, stratified by age group. The blue lines and orange lines depict the observed and predicted co-infection rates, respectively. (C) Geographical distribution of *M*. *perstans* co-infections with malaria and *L*. *loa*. (D) Geographical distribution of *L*. *loa* co-infections with malaria and *M*. *perstans*. In (C) and (D) only 1x1 km^2^ grids with at least one case of *M*. *perstans* or *L*. *loa* infection are presented. The areas marked in green are nature reserves.

Next, we wanted to estimate the proportion of malaria positive individuals co-infected with filarial nematodes at the molecular level. From the total 3214 mRDTs extracted, we analysed 2775 mRDTs for *Plasmodium* spp., by using the PlasQ assay because of limited availability of NA. Ten out of 2775 mRDTs were positive for both, *Plasmodium* spp., and *L*. *loa*, 32 were positive for both, *Plasmodium* spp., and *M*. *perstans*, and six were positive for both *M*. *perstans* and *L*. *loa*. Triple infection of *M*. *perstans*, *L*. *loa*, and *Plasmodium* spp., was found in three samples (Fig 4A). Then, we analyzed the likelihood that predicted co-infection rates between *Plasmodium* spp. and either *M*. *perstans* or *L*. *loa* differed from observed proportions indicative of biological interaction between these infectious diseases as described previously [27]. We did not find evidence for unexpected higher or lower proportions of co-infections in any of the investigated age groups (Fig 4B). Coinfections were mapped with areas positive for either *M*. *perstans* (Fig 4C) or *L*. *loa* (Fig 4D). Infections were highlighted according to the presence of people infected with more than one of the investigated parasites. Most coinfections were observed in Malabo, the area with the highest population density.

### Age is associated with variation of microfilaria levels in peripheral blood

**Fig 5:**
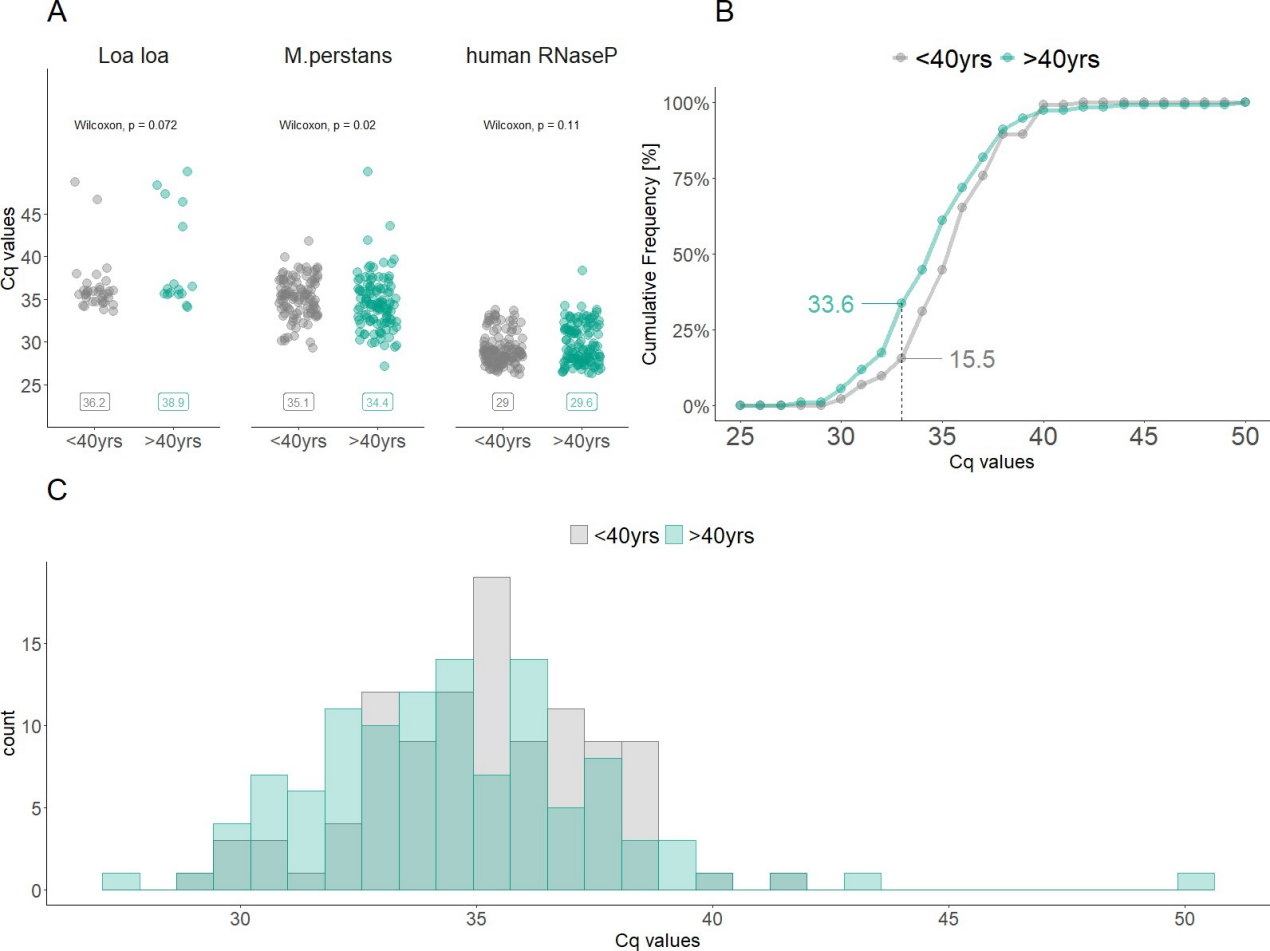
Comparison of Cq values obtained from *M*. *perstans* and *L*. *loa* infected individuals aged above and below 40 years. (A) Scatter plots of Cq values for *L*. *loa*, *M*. *perstans* qPCR and the corresponding Cq values for the human RNaseP gene qPCR. The geometric mean values for each group are shown. (B) Cumulative frequency of Cq values for *M*. *perstans* infected individuals. At a Cq value of 33 (dashed line), the cumulative frequency among individuals aged above 40 years is 33.6% compared to 15.5% among individuals aged below 40 years. (C) Histogram of the distribution of Cq values for *M*. *perstans* infected individuals stratified according to age.

The llmp-qPCR Cq values for both, *M*. *perstans* and *L*. *loa*, were used as approximations of parasite density and compared between infected individuals older and younger than 40 years of age. The median age of all *M*. *perstans* positive individuals was 40 years. Persons older than 40 years had a significantly lower *M*. *perstans* Cq values compared to individuals below the age of 40 years (geom. Mean of 34.0 versus 35.1, p = 0.02) (Fig 5A). The difference in *M*. *perstans* infection parasite densities associated with age is not the result of variation in blood volumes analysed or amount of DNA amplified since the Cq values of the human RNaseP gene is similar between the two groups. The same outcome can be observed in Fig 5B. For instance, at a Cq value of 33, the cumulative frequency of individuals older than 40 years of age is 33.6% compared to only 15.5% for younger individuals. Analysing the distribution of the *M*. *perstans* Cq values reveals a clear shift towards lower Cq values in infected individuals above the age of 40 years (Fig 5C). Combining all these findings directs towards the conclusion that older individuals have a tendency to higher *M*. *perstans* parasite densities. No significant differences were observed among the individuals infected with *L*. *loa*. Apart from age (S3A Fig), no significant difference was observed between female and male gender (S3B Fig), while the parasite density of *M*. *perstans* infections was higher in rural areas comprising districts of Baney, Luba and Riaba compared to the more urban district of Malabo (p = 0.018) (S3C Fig). Interestingly, no difference of llmp-qPCR Cq values were seen in samples collected during the morning versus afternoon (S3D Fig).

## Discussion

We conducted a larger scale, cross-sectional study of samples including paediatric, adolescent, adult and elderly populations residing in urban and rural regions of Bioko Island. To the best of our knowledge, this report represents the first molecular epidemiological study of *M*. *perstans* and *L*. *loa* in Equatorial Guinea that also includes the evaluation of geographical distribution and association with socio-economic status.

Between 1978 and 2020, a total of 20 publications described Mansonellosis, Loasis, Onchocerciasis, and lymphatic filariasis in the context of Equatorial Guinea (S1 Table). Five of these publications described *L*. *loa* and *M*. *perstans* infections among Equato-Guineans living in Spain [28–31] or Singapore [32], while three were case reports of foreigners returning from Equatorial Guinea [33–35]. One cross-sectional study investigated the prevalence of *L*. *loa* and *M*. *perstans* on Bioko Island using microscopy and qPCR-based detection [36].

We have extended our mRDT-based molecular surveillance tool, originally developed for malaria, to the filarial nematodes *M*. *perstans* and *L*. *loa*. The widespread availability and use of mRDTs in malaria endemic regions that are also endemic to *M*. *perstans* and *L*. *loa*, the simplicity of mRDT collection and storage, would make this approach convenient for large-scale molecular epidemiological studies covering *Plasmodium* spp., *M*. *perstans* and *L*. *loa*. Using our extraction protocol based on mRDTs, high quality and sufficient quantities of *M*. *perstans* and *L*. *loa* specific DNA fragments were obtained as demonstrated by successful Sanger sequencing of the ribosomal internal transcribed spacer 1 region. Therefore, apart from amplifying short DNA fragments usually used for qPCR, our NA extraction method also allows to amplify larger fragments suitable for genotyping of the pathogens of interest. In future, switching to more polymorphic markers for genotyping in combination with next generation sequencing technologies might improve tracking of infections and importantly help to understand if there are multiple strain infections that possibly accumulate over time in the elderly population showing the highest parasite density of *M*. *perstans* infections by qPCR.

The cost of our mRDT-based *M*. *perstans* and *L*. *loa* test system was estimated to be $4 per sample, from which $3 were spent for NA extraction and $1 on the llmp*-*qPCR assay. Noteworthy, the same aliquot of extracted NA was used for genomic characterization and quantification of malaria parasites as described [19] making this approach highly cost efficient. The low cost and high scalability of our approach enables systematic monitoring of impact of public health interventions against blood borne pathogens through large scale surveillance.

Here, we found that infections with *M*. *perstans* (6.6%) are more prevalent than *L*. *loa* (1.5%) and that *M*. *perstans* infections can be mostly found in the older, male, population living in rural parts of Bioko Island. This finding reconfirms previous reports [37–39]. We found similar prevalence data for *M*. *perstans* and *L*. *loa* compared to a qPCR-based cross-sectional study conducted on Bioko Island in 2018. Ta and colleagues found that 8.8% and 0.7% of persons tested were infected with *M*. *perstans* and *L*. *loa*, respectively [36]. The similar proportions found in these two independent studies indicate that detection rates for both filarial nematode species are comparable in spite of different sampling methods (dried blood recovered from mRDT versus freshly collected whole blood) and blood volumes (5 μL versus 200 μL) used. The prevalence of *L*. *loa* found in both studies could be underestimated and partially explained by the fact that 70% of infected individuals do not show mf circulation in peripheral blood, with occult infection or occasional presence of adult worms under conjunctive tissue [40].

The higher parasite density, as expressed by the qPCR’s Cq values, of *M*. *perstans* infection found in rural areas on the East coast of Bioko Island might reflect the distribution or abundance of the vector and its active transmission in those areas. The vector *Culicoides* presence is more likely associated to aquatic environments, banana and plantain stems [9] that might describe the environmental characteristics of rural areas in Bioko Island. Entomological monitoring for the presence of these vectors would be justified to improve the understanding of the geographic patterns observed and inform control interventions. Elderly people above the age of 60 were proportionally the most affected age group. The increased infection rates in combination with higher *M*. *perstans* parasite densities compared to younger people might be due to the cumulative effect of reinfection during their lifespan [39].

It has been shown that filarial worms including *M*. *perstans* and *L*. *loa* cause chronic infections that are associated with strong immune modulation in the human host [41,42]. These long- standing and strong immune modulatory effects particularly of *M*. *perstans* might negatively impact on mf clearance [37] as well as on co-infections like malaria or tuberculosis outcomes in the same host [43]. In addition, albeit not clinically overt, *M*. *perstans* infections might impair vaccine induced immune responses and protection by exerting strong immune modulatory effects as described for other helminth infections [44,45]. Therefore, molecular epidemiological studies using the methodology outlined here may prove critical in identifying cofounders of the protective efficacy of experimental malaria vaccine studies currently ongoing in Equatorial Guinea [46,47].

Quantitative measurements of filarial nematodes might become important in the context of development of novel drug interventions against Mansonellosis, loiasis, lymphatic filariasis and onchocerciasis in areas with high co-infections between these parasites [48]. The lack of an international standard with predefined numbers of mf of each of the filariasis causing parasites that could be used for quantitative assessment of microfilaremia based on Cq values measured is one of the tools limiting our approach.

Our study presented had some limitations. We restricted our analysis to *L*. *loa* and *M*. *perstans*, both blood-borne pathogens. *Onchocercha volvulus* and *M*. *stretocerca* were described on Bioko Island [36] but their mf are located in the skin and therefore are detected using primarily skin biopsies for microscopy for molecular analyses. Although Lymphatic filariasis has not been reported on Bioko Island, using mRDTs collected during daylight as a source of the blood sample would not allow exploring the presence of *Wuchereria bancrofti*. Also, we have used primers/probe combinations in our Llmp-qPCR assay that could most likely not amplify the newly identified Mansonella sp “DEUX, thereby potentially omitting this novel *Mansonella* species described recently [4]. We conducted a feasibility study to demonstrate that it is possible and sensible to use the mRDTs and metadata collected during an annual MIS to assess at very low additional costs the positivity rate of highly neglected nematode filarial infections for different demographic and socio-economic groups. However, a full analytical and clinical performance evaluation to determine the sensitivity and specificity of our approach based on a direct comparison with microscopy would be needed to fully understand the limitations of our molecular testing for routine surveillance of filarial nematodes in endemic regions.

## Conclusion

In summary, our approach of repurposing used mRDT as source of NA provides a promising, future tool that enables a cost-effective approach to monitor the prevalence, genotypes, parasite densities and co-infections of filarial nematodes and potentially other blood borne infectious diseases. Also, PCR amplification and sequencing of DNA fragments allowing for genotyping extends the range of possible applications of using NA stored on mRDTs. This method might be of particular interest in settings with limited access to cool chains, laboratory infrastructure and in populations not necessarily served by clinics and health posts in rural areas.

## Supporting information

S1 Table Literature review on studies carried out on filarial nematodes in Equatorial Guinea or Equato-Guineans living abroad. (DOCX)

S2 Table Positivity rates of *M*. *perstans* and *L*. *loa* stratified by gender, age, district and socio-economic status. (DOCX)

S1 Fig Selection of mRDTs used for NA extraction and molecular analysis stratified by mRDT result (A), age groups (B) and district (C).
(TIFF)

S2 Fig Representative amplification plots for the llmp-qPCR assay. (A) Multiplex qPCR amplification of the human RNase P gene, *M*. *perstans* and *L*. *loa*. (B) Curves in purple show amplification of the RNaseP gene used as an internal extraction and qPCR amplification control. (C) Curves in yellow show the amplification and detection of *M*. *perstans*-specific 18S target by qPCR. (D) Curves in blue show the amplification and detection of the *L*. *loa*-specific LLMF72 target by qPCR.
(TIF)

S3 Fig Variation of Llmp-qPCR Cq values for *L*. *loa* (white) and *M*. *perstans* (grey). Grouped by age (A), gender (B), urban or rural residence (C) and day time of blood collection (D).
(TIFF)

S4 Fig Graphical representation of samples selected for the Sanger Sequencing experiment of the ribosomal internal transcribed spacer 1 region. All *M*. *perstans* or *L*. *loa* positive samples, sorted by their Cq values are shown. Samples selected for Sanger Sequencing are highlighted in red.
(TIFF)
