# Supplementary material for: Characterising co-infections with Plasmodium spp., Mansonella perstans or Loa loa in asymptomatic children, adults and elderly people living on Bioko Island using nucleic acids extracted from malaria rapid diagnostic tests
Source: PLoS Negl Trop Dis. 2022 Jan 31;16(1):e0009798. doi: 10.1371/journal.pntd.0009798 (PMC8830708; doi:10.1371/journal.pntd.0009798)
Supplement: S1 Table — (DOCX) [file pntd.0009798.s001.docx]

S1 Table. Literature review on studies carried out on filarial nematodes in Equatorial Guinea or Equato-Guineans living abroad

| **Species** | **Study type** | **Population investigated** | **Findings/Prevalence** | **Diagnostic methods used** | **Location** | **Year of investigation** | **Ref.** |
| --- | --- | --- | --- | --- | --- | --- | --- |
| *L. loa* | Retrospective investigation | African immigrants with 94 % from Equatorial Guinea | 131/5700 cases  45.8 % *L .loa* only  54.2 % co-infection with (*L. loa, O. volvulus, M. streptocerca, W. bancrofti*) | - Clinical manifestation (eye visualization, eosinophilia, calabar swelling, subcutaneous lesion)  - Microscopy | Spain | 2020 | [1] |
| *M. perstans* | Retrospective investigation | African immigrants with 94 % from Equatorial Guinea | 503/5700 cases  61,2 % *M. perstans* only  38.8 % co-infection with (*L. loa, O. volvulus, M. streptocerca, W. bancrofti*) | - Clinical manifestation (eye visualization, eosinophilia, calabar swelling, subcutaneous lesion)  - Microscopy | Spain | 2020 | [2] |
| *L. loa* | Case report | Equatoguinean | One case (9 years old girl) | - Clinical manifestation  - Microscopy  - PCR | Spain | 2019 | [3] |
| *L. loa,*  *O. volvulus,*  *M. perstans* | Cross-sectional study | Equatoguinean  (543 participants) | *M. perstans* (8.8%)  *L. loa* (0.7%). | - qPCR | Bioko Island (Equatorial Guinea) | 2014 | [4] |
| *O. volvulus,*  *W. bancrofti* | Cross-sectional study | Equatoguinean  (7052 children) | *O. volvulus* and *W. bancrofti* negative by PCR | -Serological (Ov16 RDT, ELISA)  -qPCR | Bioko Island (Equatorial Guinea) | 2016-2017 | [5] |
| *O. volvulus* | Cross-sectional study | Equatoguinean  (150 households) | *O. volvulus* | -Serological (ELISA) | Bioko Island (Equatorial Guinea) | 2014 | [6] |
| *L. loa* | Case report | US national visiting Bioko Island | One case (25 years old woman) | -Clinical manifestations (swelling the ankle, knee pain, pruritis, eosinophilia)  -PCR | USA | 2016 | [7] |
| *O. volvulus* | Cross-sectional study | Equatoguinean  (544 participants) | Skin PCR result: *O. volvulus* (one), *L. Loa* (one), *M. perstans* (seven), *M. streptocerca* (two) | -Microscopy (skin snip)  -Serology test (ELISA)  -qPCR | Bioko Island (Equatorial Guinea) | 2014 | [8] |
| *O. volvulus* | Cross-sectional study | Equatoguinean  (544 participants) | *O. volvulus* (7.9 %) | -Serology test (ELISA) | Bioko Island (Equatorial Guinea) | 2014 | [9] |
| *L. loa* | Case report | Equatoguinean | One case (woman) | - Clinical manifestations (eye visualization, eosinophilia) | Equatorial Guinea | 2013 | [10] |
| *L. loa* | Case report | Chinese national visiting Equatorial Guinea | One (35 years old men) | - Clinical manifestations (swelling of the wrist, eosinophilia)  - Nested PCR | China | 2012 | [11] |
| *L. loa* | Case report | Equatoguinean | Two (23 years old man and 18-years old woman) | -Clinical manifestation (eye visualization) | Equatorial Guinea | 2010 | [12] |
| *O. volvulus,*  *L. loa,*  *M. perstans* | Retrospective investigation | Equatoguinean | 14 cases (children aged between 3 and 15 years old):  *O. volvulus* (8] , *M. perstans* (8) and *L. loa* (2) | -Clinical manifestation (pruritis, eye visualization, eosinophilia)  - Microscopy | Spain | 1995 to 2007 | [13] |
| *O. volvulus* | Cross-sectional study | Equatoguinean  (1723 individuals in 1989 and 1082 in 1998) | 77.1 % in pre-treatment (1989)  35.8 % in post-treatment (1998) | -Microscopy (skin snip) | Bioko Island | 1989-1998 | [14] |
| *L. loa* | Case report | Equatoguinean | Seven (22-43 years old men) | -Clinical manifestations (swelling of the hands and arms, itching and pain in the eyes, eye worms, eosinophilia) | Singapore | 2002 | [15] |
| *O. volvulus* | Cross-sectional study | Equatoguinean  (3218 individuals) | 6.8% with visual impairment  3.2 % with blindness | -Clinical manifestations (visual acuity) | Bioko Island (Equatorial Guinea) | 1999 | [16] |
| *L. loa* | Case report | Morrocan nationals visiting Equatorial Guinea | Twenty-four | -Clinical manifestations (pruritis, edema, eye worms) | Morocco | 2001 | [17] |
| *O. volvulus* | Cross-sectional study | Equatoguinean  (1799 participants) | 75.2 % | -Clinical manifestations (nodules, dermatitis, blindness, lymphedema)  -Microscopy (skin snips) | Bioko Island | 1987-1989 | [18] |
| *L. loa,*  *M. perstans* | Comparative study | Equatoguinean  (829 participants) | Parasitological incidence was 27.1 % for *L. Loa* and 66.3 % for *M. perstans* | -Microscopy | Equatorial Guinea mainland | 1990 | [19] |
| *O. volvulus* | Case report | US national visiting Bioko Island | One case in woman | -Microscopy | USA | 1987 | [20] |

]

**References**

1. Puente S, Ramírez-Olivencia G, Lago M, Subirats M, Bru F, Pérez-Blazquez E, et al. Loiasis in sub-Saharan migrants living in Spain with emphasis of cases from Equatorial Guinea. Infectious diseases of poverty. 2020;9(1):16.

2. Puente S, Lago M, Subirats M, Sanz-Esteban I, Arsuaga M, Vicente B, et al. Imported Mansonella perstans infection in Spain. Infectious diseases of poverty. 2020;9(1):105.

3. Placinta IA, Pascual CI, Chiarri-Toumit C, Mata-Moret L, Sanchez-Cañizal J, Barranco-González H. Ocular loiasis affecting a child and its assessment by Anterior Segment Optical Coherence Tomography. Rom J Ophthalmol. 2019;63(2):184-7.

4. Ta TH, Moya L, Nguema J, Aparicio P, Miguel-Oteo M, Cenzual G, et al. Geographical distribution and species identification of human filariasis and onchocerciasis in Bioko Island, Equatorial Guinea. Acta tropica. 2018;180:12-7.

5. Herrador Z, Garcia B, Ncogo P, Perteguer MJ, Rubio JM, Rivas E, et al. Interruption of onchocerciasis transmission in Bioko Island: Accelerating the movement from control to elimination in Equatorial Guinea. PLoS neglected tropical diseases. 2018;12(5):e0006471.

6. Gómez-Barroso D, Moya L, Herrador Z, García B, Nguema J, Ncogo P, et al. Spatial clustering of onchocerciasis in Bioko Island, Equatorial Guinea. J Infect Dev Ctries. 2018;12(11):1019-25.

7. Priest DH, Nutman TB. Loiasis in US Traveler Returning from Bioko Island, Equatorial Guinea, 2016. Emerging infectious diseases. 2017;23(1):160-2.

8. Moya L, Herrador Z, Ta-Tang TH, Rubio JM, Perteguer MJ, Hernandez-González A, et al. Evidence for Suppression of Onchocerciasis Transmission in Bioko Island, Equatorial Guinea. PLoS neglected tropical diseases. 2016;10(7):e0004829.

9. Hernández-González A, Moya L, Perteguer MJ, Herrador Z, Nguema R, Nguema J, et al. Evaluation of onchocerciasis seroprevalence in Bioko Island (Equatorial Guinea) after years of disease control programmes. Parasites & vectors. 2016;9(1):509.

10. Burgués-Ceballos A, Marcos MA, March GA, Juberías JR. [Ocular loiasis in a patient with chronic hypereosinophilia]. Archivos de la Sociedad Espanola de Oftalmologia. 2014;89(10):411-3.

11. Wang X, Zhang X, Zong Z. A Case of loiasis in a patient returning to China diagnosed by nested PCR using DNA extracted from tissue. Journal of travel medicine. 2012;19(5):314-6.

12. Lichtinger A, Caraza M, Halpert M. Subconjunctival loiasis. The American journal of tropical medicine and hygiene. 2011;84(2):183.

13. Cuello MR, Cuadros EN, Claros AM, Hortelano MG, Fontelos PM, Peña MJ. [Filarial infestation in patients emanating from endemic area. 14 cases series presentation]. Anales de pediatria (Barcelona, Spain : 2003). 2009;71(3):189-95.

14. Mas J, Ascaso C, Escaramis G, Abellana R, Duran E, Sima A, et al. Reduction in the prevalence and intensity of infection in Onchocerca volvulus microfilariae according to ethnicity and community after 8 years of ivermectin treatment on the island of Bioko, Equatorial Guinea. Tropical medicine & international health : TM & IH. 2006;11(7):1082-91.

15. Lee LS, Paton NI. Importation of seven cases of an unusual helminthic infection into Singapore and assessment of the risk of local transmission. Singapore medical journal. 2004;45(5):227-8.

16. Moser CL, Martín-Baranera M, Vega F, Draper V, Gutiérrez J, Mas J. Survey of blindness and visual impairment in Bioko, Equatorial Guinea. Br J Ophthalmol. 2002;86(3):257-60.

17. El Haouri M, Erragragui Y, Sbai M, Alioua Z, Louzi, El Mellouki W, et al. [Cutaneous filariasis Loa Loa: 26 moroccan cases of importation]. Annales de dermatologie et de venereologie. 2001;128(8-9):899-902.

18. Mas J, Yumbe A, Solé N, Capote R, Cremades T. Prevalence, geographical distribution and clinical manifestations of onchocerciasis on the Island of Bioko (Equatorial Guinea). Tropical medicine and parasitology : official organ of Deutsche Tropenmedizinische Gesellschaft and of Deutsche Gesellschaft fur Technische Zusammenarbeit (GTZ). 1995;46(1):13-8.

19. Vila Montlleo R. [Serum filariasis due to Loa loa and Mansonella perstans in the continental area of equatorial Guinea. Preliminary results]. Medecine tropicale : revue du Corps de sante colonial. 1990;50(4):399-402.

20. Joyce MP, Pearson RD. Upper extremity swelling and hyperpigmentation due to onchocerciasis in an American. Southern medical journal. 1987;80(11):1452-4.
