# Supplementary material for: Characterising co-infections with Plasmodium spp., Mansonella perstans or Loa loa in asymptomatic children, adults and elderly people living on Bioko Island using nucleic acids extracted from malaria rapid diagnostic tests
Source: PLoS Negl Trop Dis. 2022 Jan 31;16(1):e0009798. doi: 10.1371/journal.pntd.0009798 (PMC8830708; doi:10.1371/journal.pntd.0009798)
Supplement: S2 Table — (DOCX) [file pntd.0009798.s002.docx]

**S2 Table. Positivity rates of *M. perstans* and *L. loa* stratified by gender, age, district and socio-economic status.**

| Characteristics | Total  N (%) | | *M. perstans*  Positive (%) | *p-value* | | *L. loa*  Positive (%)) | *p-value* |
| --- | --- | --- | --- | --- | --- | --- | --- |
| **Gender** | | | | | | | |
| Women | 2086 (55) | 74 (3.9) | | 4.2e-16 | 26 (1.4) | | 0.4 |
| Men | 1704 (45) | 139 (10.5) | |  | 24 (1.8) | |  |
| **Age (years)** | | | | | | | |
| 0-19 | 1460 (45.4) | 32 (2.2) | | < 2.2e-16 | 8 (0.5) | | 0.0001 |
| 20-39 | 1059 (33) | 74 (7.0) | |  | 26 (2.5 ) | |  |
| 40-59 | 391 (12.2) | 58 (14.8) | |  | 8 (2.0) | |  |
| ≥ 60 | 303 (9.4) | 49 (16.2) | |  | 7 (2.3) | |  |
| **District** | | | | | | | |
| Malabo | 2064 (64.2) | 46 (2.2) | | < 2.2e-16 | 30 (1.5) | | 0.005 |
| Baney | 690 (21.5) | 91 (13.2) | |  | 5 (0.7) | |  |
| Riaba | 203 (6.3) | 32 (15.8) | |  | 8 (3.9) | |  |
| Luba | 257 (8.0) | 44 (17.1) | |  | 7 (2.7) | |  |
| **Socio-economic status (quintile)** | | | | | | | |
| 1 (lowest) | 525 (16.4) | 81 (15.4) | | 0.0005 | 16 (3.0) | | 0.07 |
| 2 | 599 (18.7) | 51 (8.5) | |  | 8 (1.3)) | |  |
| 3 | 664 (20.7) | 32 (4.8) | |  | 9 (1.4) | |  |
| 4 | 677 (21.1) | 28 (4.1) | |  | 10 (1.5) | |  |
| 5 (highest) | 737 (23) | 20 (2.7) | |  | 7 (0.9) | |  |
