# Supplementary figures and images for: Characterising co-infections with Plasmodium spp., Mansonella perstans or Loa loa in asymptomatic children, adults and elderly people living on Bioko Island using nucleic acids extracted from malaria rapid diagnostic tests
Source: PLoS Negl Trop Dis. 2022 Jan 31;16(1):e0009798. doi: 10.1371/journal.pntd.0009798 (PMC8830708; doi:10.1371/journal.pntd.0009798)

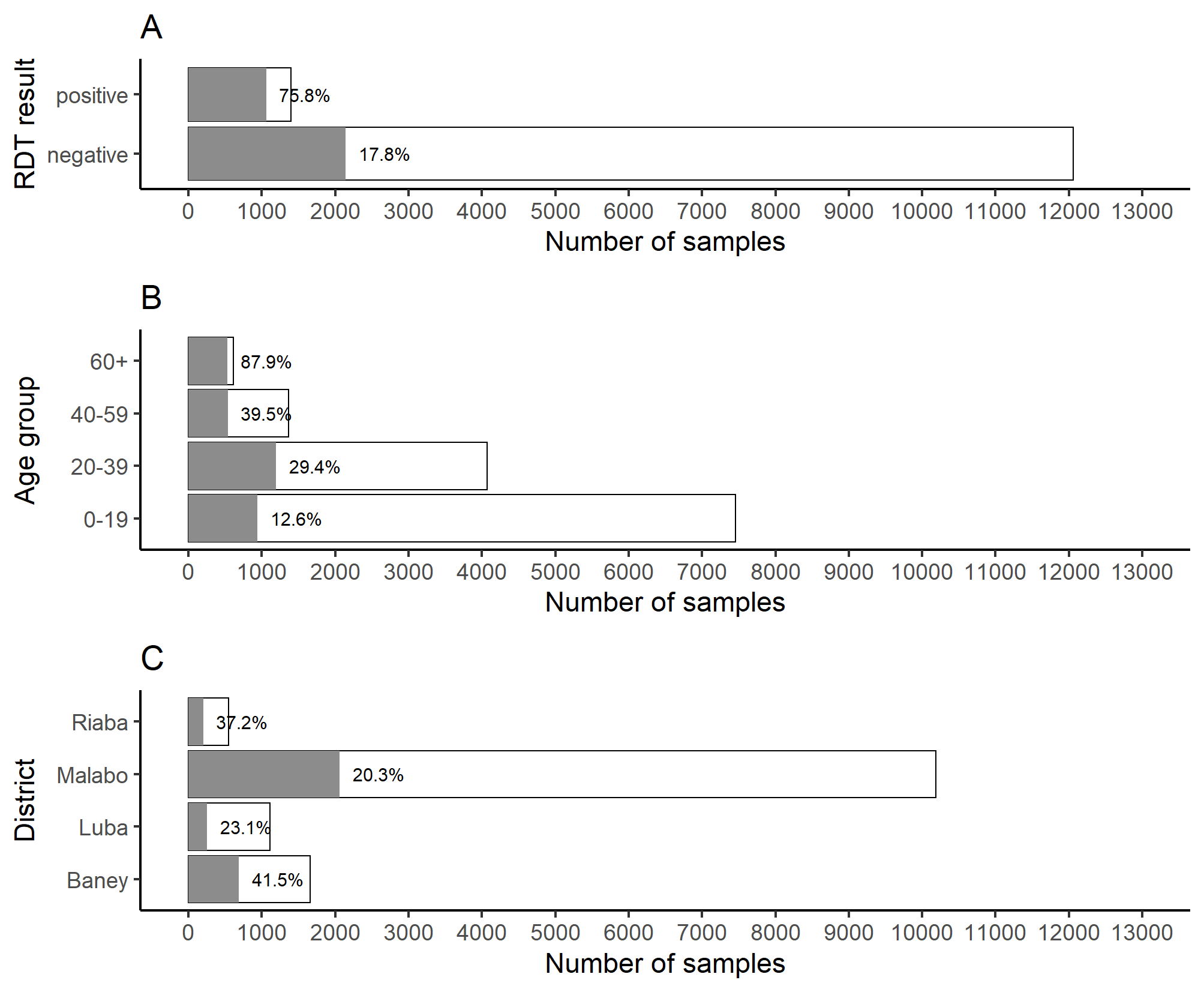

Supplement: S1 Fig — Selection of mRDTs used for NA extraction and molecular analysis stratified by mRDT result (A), age groups (B) and district (C). (TIFF) [file pntd.0009798.s003.tiff]

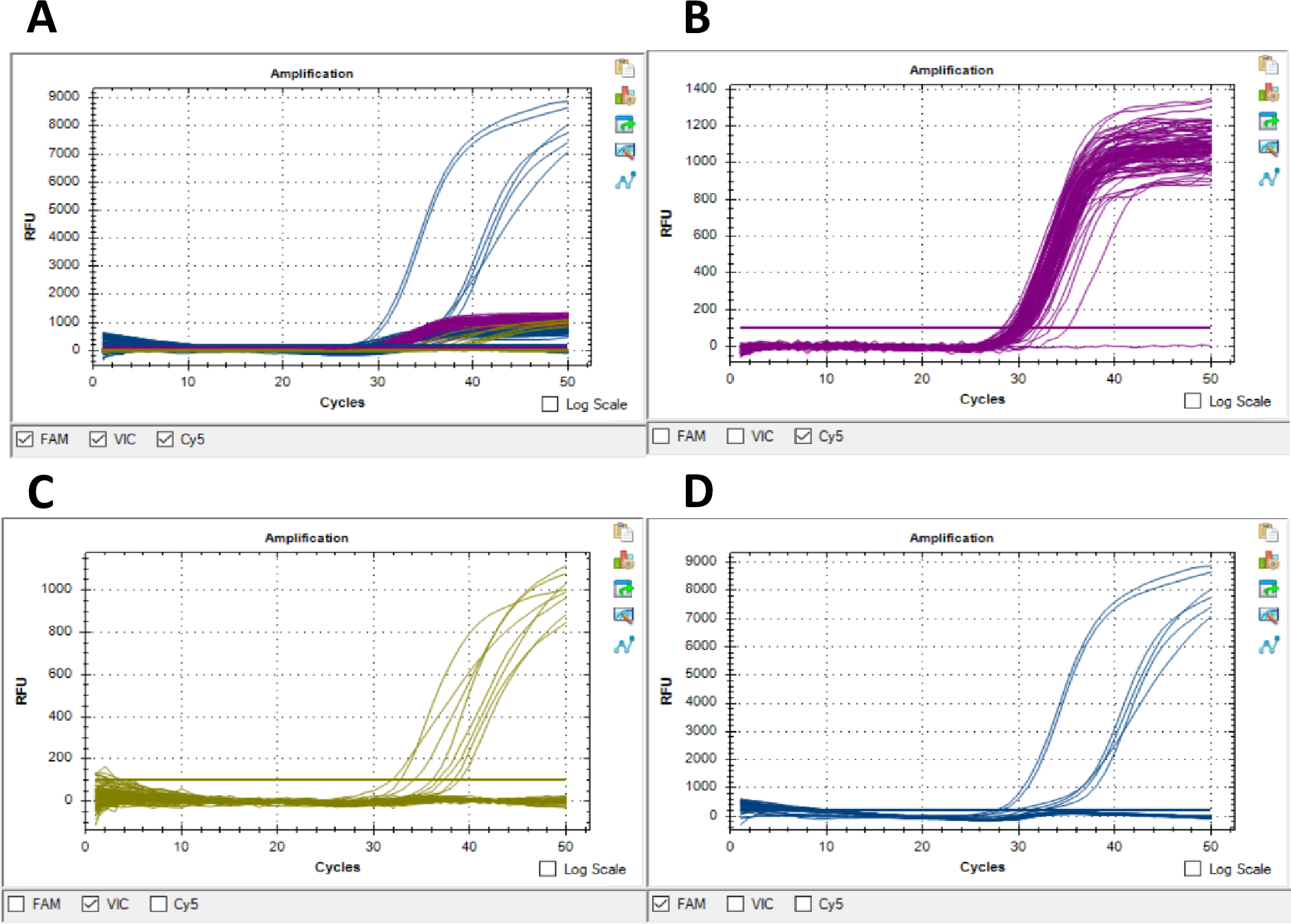

Supplement: S2 Fig — (A) Multiplex qPCR amplification of the human RNase P gene, M. perstans and L. loa. (B) Curves in purple show amplification of the RNaseP gene used as an internal extraction and qPCR amplification control. (C) Curves in yellow show the amplification and detection of M. perstans-specific 18S target by qPCR. (D) Curves in blue show the amplification and detection of the L. loa-specific LLMF72 target by qPCR. (TIF) [file pntd.0009798.s004.tif]

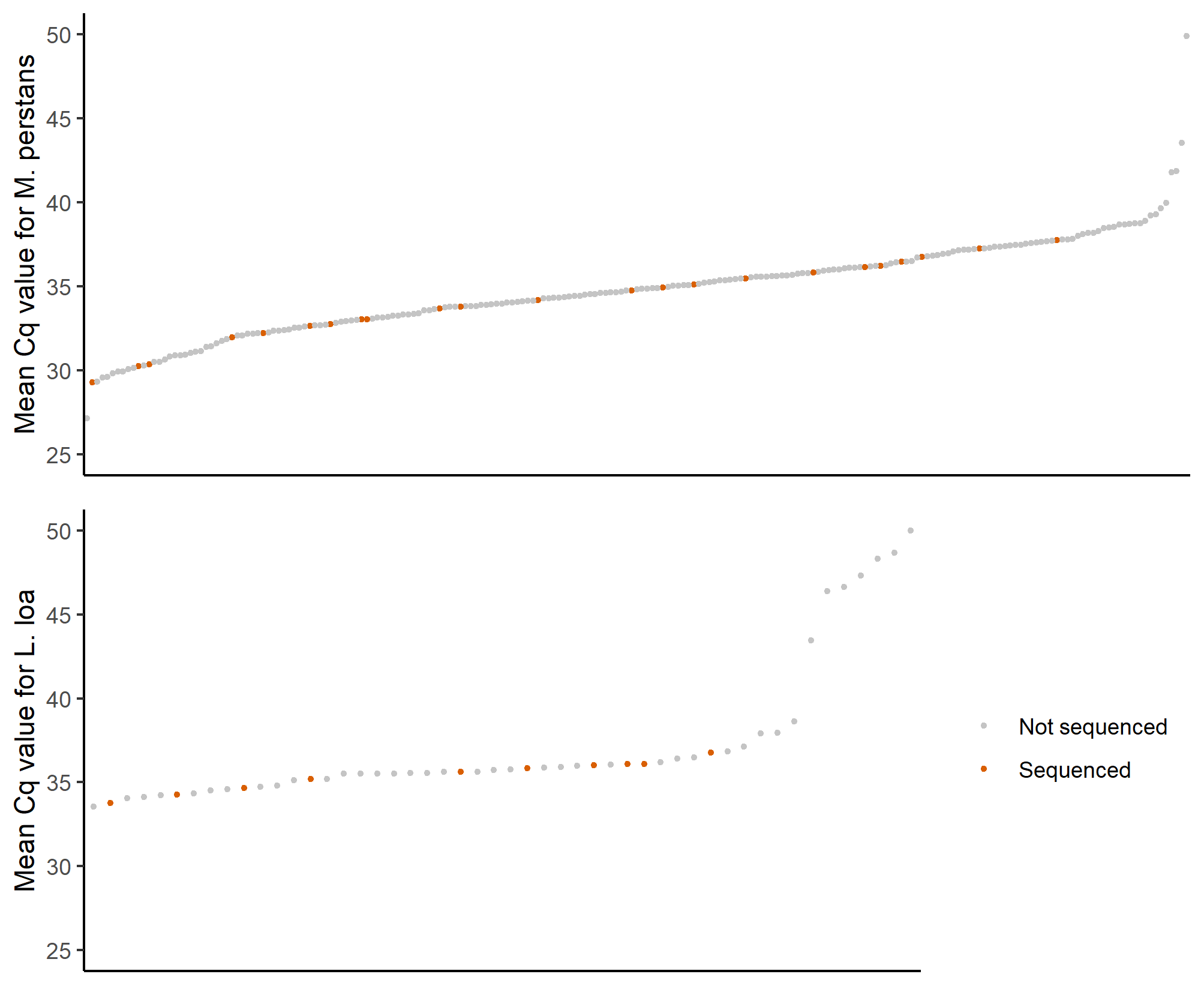

Supplement: S4 Fig — All M. perstans or L. loa positive samples, sorted by their Cq values are shown. Samples selected for Sanger Sequencing are highlighted in red. (TIFF) [file pntd.0009798.s006.tiff]
